# Eligibility for and outcome of treatment of latent tuberculosis infection in a cohort of HIV-infected people in Spain

**DOI:** 10.1186/1471-2334-10-267

**Published:** 2010-09-14

**Authors:** Asuncion Diaz, Mercedes Diez, Maria Jose Bleda, Mikel Aldamiz, Miguel Camafort, Xabier Camino, Concepcion Cepeda, Asuncion Costa, Oscar Ferrero, Paloma Geijo, Jose Antonio Iribarren, Santiago Moreno, Maria Elena Moreno, Pablo Labarga, Javier Pinilla, Joseba Portu, Federico Pulido, Carmen Rosa, Juan Miguel Santamaría, Mauricio Telenti, Luis Trapiella, Monica Trastoy, Pompeyo Viciana

**Affiliations:** 1 Unidad de Epidemiología del VIH/SIDA, Centro Nacional de Epidemiología, Instituto de Salud Carlos III, Madrid, Spain; 2 Secretaria del Plan Nacional sobre el sida, Ministerio de Sanidad y Política Social, Madrid, Spain; 3 Servicio Medicina Interna, Hospital Txagorritxu,Vitoria, Spain; 4 Servicio Medicina Interna, Hospital Mora d'Ebre, Instituto de Investigación Sanitaria "Pere Virgili", Universidad "Rovira i Virgili", Mora d'Ebre, Spain; 5 Servicio de Enfermedades Infecciosas, Hospital Ntra Sra de Aranzazu, San Sebastián, Spain; 6 Unidad VIH, Hospital Doce de Octubre, Madrid, Spain; 7 Servicio Enfermedades Infecciosas, Hospital de Basurto, Bilbao, Spain; 8 Servicio Medicina Interna, Hospital Virgen de la Luz, Cuenca, Spain; 9 Servicio de Enfermedades Infeccciosas, Hospital Ramón y Cajal, Madrid, Spain; 10 Servicio de Medicina Interna, Hospital San Millán, Logroño, Spain; 11 Unidad de Enfermedades Infecciosas, Hospital Universitario Central de Asturias, Oviedo, Spain; 12 Servicio Enfermedades Infecciosas, Hospital Virgen del Rocío, Sevilla, Spain

## Abstract

**Background:**

Previous studies have demonstrated the efficacy of treatment for latent tuberculosis infection (TLTBI) in persons infected with the human immunodeficiency virus, but few studies have investigated the operational aspects of implementing TLTBI in the co-infected population.The study objectives were to describe eligibility for TLTBI as well as treatment prescription, initiation and completion in an HIV-infected Spanish cohort and to investigate factors associated with treatment completion.

**Methods:**

Subjects were prospectively identified between 2000 and 2003 at ten HIV hospital-based clinics in Spain. Data were obtained from clinical records. Associations were measured using the odds ratio (OR) and its 95% confidence interval (95% CI).

**Results:**

A total of 1242 subjects were recruited and 846 (68.1%) were evaluated for TLTBI. Of these, 181 (21.4%) were eligible for TLTBI either because they were tuberculin skin test (TST) positive (121) or because their TST was negative/unknown but they were known contacts of a TB case or had impaired immunity (60). Of the patients eligible for TLTBI, 122 (67.4%) initiated TLTBI: 99 (81.1%) were treated with isoniazid for 6, 9 or 12 months; and 23 (18.9%) with short-course regimens including rifampin plus isoniazid and/or pyrazinamide. In total, 70 patients (57.4%) completed treatment, 39 (32.0%) defaulted, 7 (5.7%) interrupted treatment due to adverse effects, 2 developed TB, 2 died, and 2 moved away. Treatment completion was associated with having acquired HIV infection through heterosexual sex as compared to intravenous drug use (OR:4.6; 95% CI:1.4-14.7) and with having taken rifampin and pyrazinamide for 2 months as compared to isoniazid for 9 months (OR:8.3; 95% CI:2.7-24.9).

**Conclusions:**

A minority of HIV-infected patients eligible for TLTBI actually starts and completes a course of treatment. Obstacles to successful implementation of this intervention need to be addressed.

## Background

In Spain, there is an extensive overlap between the epidemiology of human immunodeficiency (HIV) and *Mycobacterium tuberculosis *(*M. tuberculosis*) infections [1]. The introduction of highly active antiretroviral treatment (HAART) has led to a reduced incidence of tuberculosis (TB) among HIV positive people, but the risk of developing TB remains much higher among this group [2] than among those without HIV infection. Thus, as suggested by studies performed in both high and low prevalence areas [3-5], treatment of latent tuberculosis infection (TLTBI) should be used in combination with HAART to control TB in this population.

The efficacy of TLTBI in people co-infected with HIV and TB has been extensively demonstrated [6,7], but no treatment will have a demonstrable effect on public health if the target population is not reached or if treatment compliance is far from optimal, as seems to be the case with TLTBI [8,9].

Although HIV is the main risk factor for developing TB, few studies have investigated the operational aspects of implementing TLTBI in the co-infected population [10], and none has been performed in Spain. For this reason we carried out a 3-year longitudinal study to describe tuberculin skin testing (TST) and the prevalence of *M. tuberculosis *in HIV-infected people in Spain and to investigate obstacles to successful completion of TLTBI in the co-infected population.

A cohort of 1242 HIV-infected subjects was identified during the study period, of whom 84 had past TB, 23 had current TB, and 87 had already been treated for LTBI at study entry. Thus, only 1048 persons were eligible for TST, of whom 853 (81.4%) underwent testing. The reasons for not undergoing TST were: test not prescribed by the physician (185 cases), patient's refusal (7 cases), and unknown (3 cases). A detailed account of reasons for non-prescription of TST and of *M. tuberculosis *prevalence in this cohort has already been published [11,12].

The objective of this paper is to describe eligibility for TLTBI, treatment prescription, initiation and completion in HIV-infected subjects in Spain, and factors associated with treatment completion. Since our aim was to assess "routine clinical practice", no attempt was made to modify it in any way.

## Methods

A cohort of HIV-infected people not previously followed at an HIV clinic was prospectively identified between March 2000 and February 2003 at 10 Spanish hospital-based HIV clinics scattered across a wide geographical area. In Spain, HIV care is always hospital based, and antiretroviral treatment is not available outside this setting.

Subjects were eligible for the study if 1) this was their first visit to an HIV specialist, or 2) this was not the first time an HIV specialist had examined them, but they had not attended an HIV clinic in the preceding 2 years. To avoid the inadvertent inclusion of people who had been followed up in HIV clinics not included in the study, people taking antiretroviral drugs or notified as AIDS cases before study entry were excluded. A more detailed description of the cohort can be found elsewhere [12].

While guidelines for TB prevention in HIV-infected people in Spain have been issued [13], the treating physicians, in accordance with their routine clinical practice, made their own decisions about treatment eligibility, regimens chosen for TLTBI and the number of follow-up visits.

At study entry a physical examination was performed by the visiting physician. TST was performed in accordance with Spanish guidelines for HIV-positive people [14].

All subjects without signs or symptoms of disease and who had a positive Mantoux test at study entry or a documented positive TST in the past were classified as having latent tuberculosis infection (LTBI).

In accordance with Spanish guidelines [13] a person was considered to be a candidate for TLTBI if he/she did not have past/current TB and: a) was TST (+) and had not taken TLTBI previously; b) was TST (-) but was a known contact of a TB patient; or c) was TST (-) but had a CD4 count of less than 500 cells/μL, anergy was suspected and the treating physician considered that TLTBI was necessary.

Treatment outcome was classified in the following mutually exclusive categories: a) *treatment completed*: a person who, in the clinician's judgment, has completed treatment; b) *treatment interruption due to adverse effects*: a person who, on the physician's advice, interrupted treatment due to adverse effects; c) *treatment default*: a person who interrupted treatment for reasons other than adverse effects; d) *development of TB*: a person who developed TB while in TLTBI; e) *moved away*: a person who changed residence while in TLTBI and whose outcome was unknown; f) *death: *a person who died while in TLTBI independently of the cause.

By consensus among the participating clinicians, a patient was considered to be adherent if he/she had taken at least 80% of the total pills prescribed; however, treatment adherence was assessed only in accordance with the treating physician's judgment.

Socio-demographic, epidemiological and clinical information and data on follow-up visits and treatment outcome were obtained from the clinical records.

Characteristics of subjects who were prescribed TLTBI, those who initiated treatment, and those who completed it were described. The chi-squared or Fisher's exact tests were used to evaluate the association between qualitative variables. The odds ratio and its 95% confidence interval (OR, 95% CI) were used to measure the association between different variables and completion of TLTBI. The multivariate analysis was performed using logistic regression following Hosmer and Lemeshow model-building strategies and methods [15], taking into consideration that the sample size was not very large [16]. Data analysis was performed using the STATA program (Version 10.0).

The study was performed following all the requirements of the Spanish legislation on data protection in effect at the time, and was reviewed by the corresponding boards of the institutions involved.

## Results

### a) Eligibility for TLTBI and treatment initiation

**Figure 1:**
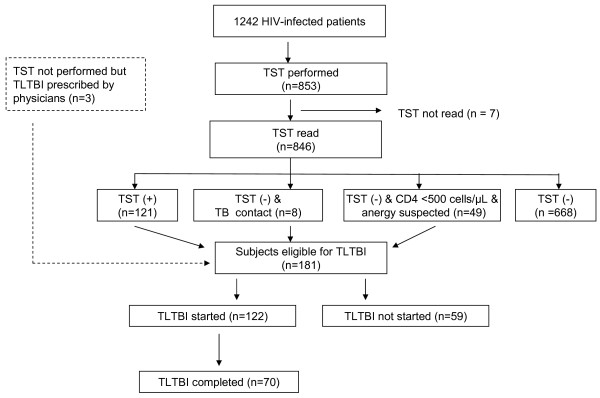
**Eligibility for treatment of latent tuberculosis infection, treatment initiation and result**. TST: tuberculin skin testing; TLTBI: treatment of latent tuberculosis infection

Of the 853 HIV-infected subjects who underwent TST, 846 (99.2%) returned for the reading, and 121 (14.3% of the tests read) were TST(+); none of them had signs or symptoms of TB disease and were, thus, eligible for TLTBI. Additionally, the treating physicians considered that 57 TST(-) and three TST unknown patients were also eligible for TLTB (Figure 1). Thus, of the 856 HIV-positive people evaluated for TLTBI, 181 (21.1%) were found to be eligible. However, only 122 of the latter (67.4%) finally initiated treatment (Figure 1).

Reasons for not initiating treatment were: a) physicians questioned patients' compliance due to their social situation (n = 20); b) medical contraindication (n = 19); c) patient's refusal (n = 17); and d) unknown causes (n = 3). All patients who rejected TLTBI were Spaniards: 13 were intravenous drug users (IDU) and were infected with Hepatitis C virus (HCV), 9 were unemployed, and 8 had poor or no education. Characteristics of subjects eligible for treatment of LTBI and those who actually initiated it are presented in table 1.

**Table 1:** Characteristics of study subjects according to treatment of latent tuberculosis infection

Variables	Eligible for TLTBI		Initiated TLTBI		Completed TLTBI	
	
	N	(%)	n	(%)	n	(%)
Sex						
Male	141	(77.9)	92	(75.4)	52	(74.3)
Female	40	(22.1)	30	(24.6)	18	(25.7)

Age group (years)						
≤ 30	48	(26.5)	39	(32.0)	20	(28.6)
31-35	53	(29.3)	33	(27.0)	15	(21.4)
36-40	40	(22.1)	22	(18.0)	13	(18.6)
≥ 41	40	(22.1)	28	(23.0)	22	(31.4)

Country of origin						
Spain	154	(85.1)	101	(82.8)	58	(82.9)
Other	27	(14.9)	21	(17.2)	12	(17.1)

Educational level						
None/incomplete primary education	24	(13.3)	14	(11.5)	6	(8.6)
Primary education completed	80	(44.2)	54	(44.3)	33	(47.1)
Secondary/university education completed	48	(26.5)	34	(27.9)	18	(25.7)
Unknown	29	(16.0)	20	(16.3)	13	(18.6)

Employment status						
Employed	73	(40.3)	49	(40.2)	33	(47.1)
Unemployed	83	(45.9)	56	(45.9)	27	(38.6)
Other	20	(11.0)	13	(10.6)	7	(10.0)
Unknown	5	(2.8)	4	(3.3)	3	(4.3)

HIV transmission route						
IDU	101	(55.8)	59	(48.4)	28	(40.0)
MSM	22	(12.2)	17	(13.9)	7	(10.0)
Heterosexual sex	53	(29.3)	44	(36.1)	34	(48.6)
Other/unknown	5	( 2.8)	2	(1.6)	1	(1.4)

Viral load (copies)						
< 50	17	(9.4)	8	(6.6)	6	(8.6)
50-9,999	57	(31.5)	43	(35.2)	29	(41.4)
10,000-54,999	45	(24.9)	30	(24.6)	15	(21.4)
≥ 55,000	58	(32.0)	39	(32.0)	19	(27.1)
Unknown	4	(2.2)	2	(1.6)	1	(1.4)

CD4 (cells/μL)						
< 100	22	(12.2)	11	(9.0)	5	(7.1)
100-199	24	(13.3)	16	(13.1)	10	(14.3)
200-499	75	(41.4)	52	(42.6)	28	(40.0)
≥ 500	60	(33.1)	43	(35.2)	27	(38.6)

Hepatitis B carriers						
Yes	14	(7.7)	9	(7.4)	3	(4.3)
No	163	(90.1)	112	(91.8)	67	(95.7)
Unknown	4	(2.2)	1	(0.8)	0	(0.0)

Hepatitis C virus antibodies						
Yes	107	(59.1)	62	(50.8)	31	(44.3)
No	69	(38.1)	57	(46.7)	38	(54.3)
Unknown	5	(2.8)	3	(2.5)	1	(1.4)

**TOTAL**	**181**	**(100.0)**	**122**	**(100.0)**	**70**	**(100.0)**

TLTBI: treatment of latent tuberculosis infection; IDU: Intravenous drug user; MSM: Men who have sex with men

The regimens prescribed for TLTBI were: a) isoniazid 6 months (6H): 51 patients (41.8%); b) isoniazid 9 months (9H): 26 (21.3%); c) isoniazid 12 months (12H): 22 (18.0%); d) rifampin plus pyrazinamide 2 months (2RZ): 17 (13.9%); e) rifampin plus isoniazid 3 months (3RH): 5 (4.1%); and f) rifampin plus isoniazid plus pyrazinamide 3 months (3RHZ): 1 patient (0.8%).

### b) Treatment outcomes

Of the 122 patients who initiated TLTBI, 70 (57.4%) completed treatment, 39 (32.0%) defaulted, 7 (5.7%) interrupted treatment because of adverse effects, 2 (1.6%) developed TB, 2 (1.6%) died, and 2 (1.6%) changed residence and the final result was unknown.

Default rates were greater among IDUs than among men who have sex with men (MSM) and heterosexuals: 44.0%, 23.5% and 18.1% respectively (p = 0.02).

Regarding treatment regimens, default rates for the long-term regimens 12 H, 6 H, or 9 H were 40.9%, 39.2% and 30.8%, respectively. The corresponding figures for the short-term regimens 2RZ, 3RH and 3RHZ were 5.9%, 20% and 0%.

Out of the 39 defaulters, 23 (59%) abandoned treatment in the first month, 16 of whom did not return after the visit when TLTBI was prescribed (Table 2).

**Table 2:** Number of defaulters, by month when treatment of latent tuberculosis infection was abandoned and type of regimen

Type of TLTBI	No of people starting TLTBI	Month Defaulting													Early defaulters/Total defaulters (%)
			
		0*	1	2	3	4	5	6	7	8	9	10	11	12	
**6H**	51	6	5	5		2	2								6/20 (30.0%)

**9H**	26	3		2		2				1					3/8 (37.5%)

**12H**	22	6	1	1							1				6/9 (66.6%)

**2RZ**	17	1													1/1

**3RH**	5		1												0/1

**Total**		**16**	**7**	**8**		**4**	**2**			**1**	**1**				**39**

TLTBI: Treatment of latent tuberculosis infection; H: isoniazid; R: rifampin; Z: pyrazinamide

* Patient did not return after the visit when TLTBI was prescribed

Out of the seven patients with adverse reactions, five developed hepatotoxicity within the first 2 months of treatment (one for each regime except 3RHZ), one developed exanthema in the fourth month of a 9 H regimen, and the last one developed gastric intolerance in the tenth month of a 12 H regimen.

The two subjects who developed TB were MSM under age 35 whose infection was detected within the first month after treatment initiation.

Two patients died while in treatment. One was an IDU co-infected with HCV who died in the first month of TLTBI and the other was a MSM with a very low CD4 count who died during the second month. Neither of these deaths was related to TLTBI.

### Factors associated with treatment completion

The 70 patients completing treatment had a mean age of 37.1 (SD:8.8) years, and were mostly men (74.3%) and Spaniards (82.9%). Almost 60.0% had acquired HIV infection through sex. Regarding their initial clinical evaluation, 50.0% had less than 10,000 viral copies, and almost 79% had more than 200 CD4 (Table 1).

Treatment completion was significantly greater among people infected with HIV through heterosexual sex than among IDU or MSM (77.3% vs. 47.5% and 41.2% respectively) (p = 0.01).

The only patient treated with 3RHZ, as well as three out of the five treated with 3RH finished the treatment. Among people treated with 2RZ the completion rate was 88.2%, and among those who had taken 9 H, 6 H or 12 H, treatment completion rates were 53.8%, 56.9% and 36.4% respectively.

The multivariate analysis to identify factors associated with treatment completion excluded subjects who died while in TLTBI, those who developed TB and those who left treatment because of adverse effects, since these persons could not complete treatment. Thus, only the 111 patients who had completed treatment, defaulted or moved away were included in this analysis. Treatment completion was greater among patients acquiring HIV through heterosexual sex as compared to IDUs (OR:4.6; 95% CI: 1.4-14.7) and those treated with 2RZ as compared to those treated with 9 H (OR:8.3; 95% CI:2.7-24.9) (Table 3).

**Table 3:** Factors associated with completed TLTBI. Univariate and multivariate analyses (n = 111)

	Univariate Analysis			Multivariate Analysis		
**Variables**	**Crude** **OR**	**(95% CI)**	**p value**	**Adjusted OR**	**(95% CI)**	**P value**

Sex						
Female	1			1		
Male	1.2	(0.9-1.6)	0.244	2.1	(0.9-4.9)	0.081

Age group (years)						
19-29	1			1		
30-34	1.1	(0.4-2.9)	0.808	1.2	(0.3-5.1)	0.789
35-38	1.2	(0.4-3.5)	0.702	1.4	0.4-4.5)	0.599
39-61	3.6	(1.1-11.9)	0.035	2.9	(0.6-13.1)	0.173

HIV transmission route						
IDU	1			1		
MSM	1.7	(0.2-12.3)	0.606	2.0	(0.3-12.2)	0.458
Heterosexual	3.6	(1.3-9.8)	0.010	4.6	(1.4-14.7)	0.010
Other/Unknown	1.0	(0.1-13.7)	0.979	1.3	(0.2-7.6)	0.793

Type of TLTBI						
9H	1			1		
6H	0.9	(0.3-2.9)	0.861	0.6	(0.2-2.2)	0.505
12H	0.6	(1.1-2.2)	0.423	0.6	(0.2-1.9)	0.382
2RZ	9.6	(1.1-81.4)	0.037	8.3	(2.7-24.9)	0.000
3RH	1.9	(0.2-21.1)	0.591	1.7	(0.1-24.2)	0.702

OR: Odds ratio; CI: confidence interval

TLTBI: Treatment of latent tuberculosis infection; IDU: Intravenous drug user; MSM: Men who have sex with men; H: isoniazid; R: rifampin; Z: pyrazinamide

Multivariate model adjusted by hospital

## Discussion

This is the first study performed in Spain that describes eligibility, initiation and outcome of TLTBI in routine clinical practice among HIV-infected subjects. In total, 1242 people entered the study. Of these, 181 were eligible for treatment, 122 initiated it, and only 70 completed treatment. Thus, only 38.7% of people eligible for TLTBI, actually finished the treatment. These findings suggest that there are many obstacles to completing TLTBI in the HIV-infected population.

Around one in five persons eligible for TLTBI did not initiate it, either because the treatment was not prescribed or because the patient refused to take it. In both instances the patients' social context seemed to be a major determinant. Thus, some physicians did not prescribe TLTBI because they expected the patient's social situation to make treatment compliance difficult; similarly, most of the patients refusing treatment had unfavorable social indicators.

Almost 11% of those potentially eligible for TLTBI did not initiate it because of medical contraindications. This figure is lower than the 17% found in a similar study performed in Italy [10], although in both studies hepatitis C was the most common contraindication.

In this study, 6% of people who began TLTBI had to interrupt it due to adverse effects. This proportion was higher than the 3.7% found in an American study where a 2RZ regimen was used [17], and lower that the 10% found in the aforementioned Italian study, where 6H/12 H regimens were used [10]. Results from other Spanish studies-two clinical trials in HIV-infected people and an observational study in non-HIV infected people [18-20]-showed higher rates of adverse effects: 15%, 9.7% and 12.4%, respectively. These differences may be due to different regimens being used or to closer patient follow-up.

The proportion of defaulters in our study was higher than the 22% and 27% found in the Spanish clinical trials [18,19]. Defaulter rates in other non-Spanish observational studies performed in HIV-infected patients [10,17,21] vary considerably, ranging from 8% to 42%, depending on the type of regimen chosen and whether or not directly observed treatment (DOT) was used.

Defaulting was common with long-term regimens, but it is interesting to note that most patients abandoned early in the course of treatment, and more than 40% of the defaulters failed to return for the first follow-up visit. This finding, common to other studies [17,22], may indicate that patients do not clearly understand the benefits of TLTBI.

The treatment completion rate, 57.4%, is comparable to those found in the Spanish clinical trials, 61% and 63.3% respectively [18,19]. As compared with findings from international studies, the completion rate was similar to the 61% found in an observational study carried out in United States with a 12 H regimen [17], but worse than those in two other studies in Italy and Brazil, which reported completion rates of 72.5% and 76.1, using 6-12 H and 6 H regimes, respectively [10,3].

The finding that treatment completion was better in patients who had acquired HIV through heterosexual sex than among IDUs was not at all surprising, since Spanish IDUs have also been found to have poor compliance with antiretroviral treatment [23]. In fact, what is surprising is that almost 50% of all IDUs initiating TLTBI completed it, a figure similar to what has been found in studies where DOT was used [24,25]. This "good" result is explained by the positive selection that had taken place among the IDUs during the clinical process previous to treatment prescription: IDUs were over-represented among subjects who did not undergo TST [11] and constituted the majority of eligible patients for whom TLTBI was not prescribed (82.6%).

Treatment completion was significantly better with the 2RZ regimen than with the 9 H regimen. This result is consistent with those of some clinical trials where adherence to this regimen was between 73.4% and 80.0% [26-28]. In an observational study with this regimen where DOT was utilized, the completion rate reached 93% [17]. Unfortunately, this regimen has been associated with severe liver injury, mostly in HIV-negative but also in HIV-infected people, and some institutions have withdrawn it from the list of recommended regimens for the treatment of LTBI [29]. We found no differences in the occurrence of adverse events with the different regimens, but numbers were small.

Our study has some limitations. The small number of study participants prevented an in depth analysis of factors associated with treatment prescription and treatment initiation. Such analyses would have provided a better understanding of physician-and patient-related variables associated with treatment prescription or treatment initiation. Furthermore, small sample sizes could have sometimes resulted in less precise estimates.

## Conclusions

This study shows that, despite the high prevalence of *M. tuberculosis *and TB disease in the HIV-infected population in Spain [12] and the potential benefit of treating LTBI [30], the outcome of TLTBI in routine clinical practice is far from desirable: less than half of the patients eligible for treatment actually take it. Reasons for this situation are diverse, but social or structural causes seem to play a far greater role than medical reasons.

The study results have some public health implications: a) adequate enablers should be used to facilitate TLTBI among IDUs; b) health staff should try to help patients understand the importance of TLTBI; and c) further research is needed to fully understand patient and physician behavioral factors influencing initiation and completion of TLTBI.

## Competing interests

The authors declare that they have no competing interests.

## Authors' contributions

AD worked on data collection and management, carried out the epidemiological analysis, prepared the figure and most of the tables, wrote the first version of the manuscript and contributed to all successive versions. MD was the main study researcher. She had the original idea, developed the study protocol, supervised field work and data collection, and wrote the statistical analysis plan and the final version of the manuscript. MJB performed data collection and management, quality control and statistical analysis, and reviewed all the manuscript drafts. MA, MC, XC, CC, AC, OF, PG, JAI, SM, MEM, PL, JP, JP, FP, CR, JMS, MT, LT,MT and PV were the physicians responsible for patient recruitment and follow-up in the participating hospitals. They all participated in development of the study protocol, collection of epidemiological and clinical data, and critical review of all versions of the manuscript.

## Pre-publication history

The pre-publication history for this paper can be accessed here:

http://www.biomedcentral.com/1471-2334/10/267/prepub
